# Assessing the impacts of daily Cannabis versus alcohol and methamphetamines on young Australians in youth AOD treatment

**DOI:** 10.1186/s12888-019-2403-1

**Published:** 2019-12-23

**Authors:** Amy C. Reichelt, James C. Collett, Ora Landmann, Karen T. Hallam

**Affiliations:** 1 Florey Institute of Neuroscience and Mental Health, Melbourne, Australia; 2 RMIT University, Melbourne, Australia; 3 Youth Support and Advocacy Service, Melbourne, Australia; 4 The University of Melbourne, Melbourne, Australia

**Keywords:** Cannabis, Substance abuse, Youth, AOD services, Methamphetamines, Alcohol, Psychosocial

## Abstract

**Background:**

Cannabis is the most widely used illicit substance by Australian young people, including those engaged with youth alcohol and other drug (AOD) systems. While recreational cannabis use in young people may be a developmental activity for some, for others, this usage becomes regular and be associated with poorer long term outcomes. This study reports on the rates of cannabis use and co-existing psychosocial complexity factors in the Youth Needs Census (2013 and 2016) where workers report on all clients in the youth AOD system, a cohort considered highly vulnerable.

**Methods:**

Data was examined for two rounds of data collection for the Youth Needs Census, including 823 youth AOD service engaged young people in 2016 and 1000 AOD service engaged young people in 2013, to identify usage rates, psychosocial outcomes, and changes over time.

**Results:**

Daily use of cannabis alone significantly exceeded daily usage rates for methamphetamines, alcohol, and cannabis used alongside other substances. Daily cannabis use was significantly associated with mental health problems, employment problems, education problems, family problems, and housing problems. Daily cannabis use was associated with most psychosocial complexity factors to the same extent as daily methamphetamine use and daily alcohol use, with daily cannabis users only showing lower incidence of the drug-related harm measure. Notably, daily cannabis use also increased from 2013 (47.5%) to 2016 (54.2%).

**Conclusions:**

It is imperative that the number of individuals using cannabis is considered alongside the severity of harm when assessing the social impact of this substance. Within cannabis users engaged with the youth AOD system, who often have high levels of psychosocial complexity, cannabis is used daily by a large proportion of these youths and may play a role in negatively impacting their lives.

## Background

In the Australian Institute of Health and Welfare (AIHW) National Drug Strategy Household Survey (NDSHS) 2013 [1], cannabis was found to be the most prevalent illicit drug used, with 35% of people aged 14 or older reporting lifetime use while 10.2% of people had used cannabis in the previous 12 months. Cannabis usage was most prevalent in people aged between 14 and 29, and of those in this age range who used cannabis, 45% had used at least monthly, making cannabis the most frequently used drug [1]. The increasing use of cannabis over time is being debated, with some older data indicating rises in use (9.1% of people over 14 years of age in 2007 to 10.3% in 2010) [1] whilst other data suggests relatively stable use in Australia, if not declining use for younger age groups [2].

Evidence indicates that a complex range of social, family, peer, and personal elements influence the substance use patterns of adolescents and young adults [3]. Cumulative exposure to violence in the family, school, and community significantly increases the risk of both alcohol and cannabis use in young people [4], and evidence indicates that psychosocial disadvantage may both be a risk factor for cannabis use specifically, as well as an outcome in those who commence use at an early age (prior to 16) [5]. Within the home, evidence indicates that family instability and parental substance use increase the chance of engaging in cannabis use in adolescents [6]. In relation to psychological coping and cannabis use, Hyman and Sinah [7] highlighted the relationship between the experience of stress, negative life events and trauma and cannabis use. Moreover, this research team indicated that whilst many individuals will use cannabis recreationally, a subset of people will develop chronic issues to manage chronic stress as a coping method. Traumatic experiences such as physical and sexual abuse in young people may place an individual at increased vulnerability to cannabis use to cope with negative feelings [8]. Likewise, structural factors such as housing instability may increase both use and risky behaviour associated with use [9]. Evidence from a longitudinal study in Christchurch, New Zealand highlights the association between cannabis use and worsened employment and educational attainment [5]. Hall [10] indicated that regular cannabis use doubles the risk for early school leaving and increases risks of mental health concerns. While many individuals experiment with cannabis use, evidence indicates that chronic users in particular are at high risk of unemployment in later life after controlling for psychosocial complexity covariates [11]. Overall, data from numerous participant pools indicate both complex psychosocial precipitants and outcomes of heavy use, chronic use, or early age of use onset.

Age of first use of cannabis is proposed to be an important factor in determining progression to heavy or problematic use [12–15]. Adolescence is a critical nexus where fundamental stages of neurological development and refinement occur alongside physical, social, and emotional development [16, 17]. Emerging data indicates that most mental health issues are associated with deviation from normal healthy developmental trajectories [18]. Even subtle changes in brain development during these time windows may occur due to substance use, and contribute to functional changes that persist throughout life [19, 20]. As such, adolescence is a high-risk period when a number of psychiatric and pathophysiological issues can arise, including major depressive disorder, bipolar disorder, anxiety disorders and schizophrenia [21]. Cannabis use in adolescence has specifically been associated with a range of negative impacts to mental health and psychosocial development for those with underlying vulnerability [22], especially in those predisposed to psychosis [23–26].

Whilst many occasional users have no long term consequences of cannabis use, evidence indicates poorer outcomes in heavy users. The associated functional impacts of regular cannabis use may be significant [27, 28]. Research indicates heavy adolescent cannabis users show persistent and enduring neurocognitive deficits in learning and memory related to heavy cannabis use [29], and these are more pronounced in comparison to adults [30]. These cognitive changes may be associated with psychosocial development difficulties, including disengagement with education and/or employment [5]. In Australia, young people with concerns over alcohol and/or substance use are referred to the youth alcohol and other drug (AOD) services/system to support them to manage their substance use, reduce harms and make connections with services and supports to foster psychosocial resilience. Young people engage with the Youth AOD system through self or other-referral when they (or others) consider substance use to be having a detrimental impact upon their lives. AOD services include counselling, case management, youth work, group work, residential and home detoxification programs and long residential rehabilitation facilities, with many young people getting a mix of these interventions. These young people may be disengaged from work and education, lack stable accommodation, and have significant psychosocial complexity compared with many other young people. Because of these factors, they are often under-represented in large scale government surveying and research into the psychosocial concomitants of substance use in this group is under-represented [31].

The aim of the present study was to examine the prevalence of cannabis use in young people engaged with youth (AOD) services, and to investigate the psychosocial complexity factors present in this cohort, including how they have changed over time. Methamphetamine use and alcohol use were analysed for comparison with cannabis use. Five hypotheses were formed based on AIHW usage data from the general population and empirical findings: (1) Cannabis would be more commonly and frequently used than other substances; (2) Daily cannabis use would be associated with mental health problems, drug-related harm, suicidality, employment problems, education problems, criminality, family problems, and housing problems; (3) These psychosocial complexity factors would be associated with daily cannabis use to a similar or greater extent than daily methamphetamine or alcohol use; (4) Daily cannabis use would have increased in frequency between the 2013 and 2016 census periods; and (5) The psychosocial complexity factors associated with daily cannabis use would have increased in frequency between 2013 and 2016.

## Method

### Participants

The Youth Needs Census (ThYNC) was collected in 2013 and 2016 across Victorian youth AOD services. The 2013 sample collected surveys on 1000 young people and the 2016 collection reported on a further 823 young people. In 2013, the average age of the sample was 18.93 years (*SD* = 2.82 years), with ages ranging between 8 and 27 years. Only 3 individuals were over 25 years of age. The sample included 339 females, 665 males and 6 transsexual young people. The 2016 sample was characterized by an average age of 18.84 years (*SD* = 2.74 years), with ages ranging between 10 and 27 years. Notably, only 5 individuals were over 25 years of age. The sample included 294 females, 522 males, 5 transsexual young people and 2 individuals who did not nominate a gender identity (an option available in the latter census).

### Materials

ThYNC was comprised of 56 items relating to substances used (in past month, daily or almost daily, substance of concern, substance of treatment focus, substance dependence, etc.). This was followed by an assessment of service utilization (programs used, length of service, etc). The survey then went on to review demographic and psychosocial complexity issues experienced by the young person. These complexity factors in these young people included: (i) mental health problems (including non-suicidal self-injury and suicide attempts); (ii) drug-related harm (e.g., physical injury); (iii) engagement in employment or education; (iv) criminal and forensic issues (excluding criminal offences directly resulting from drug possession and distribution); (v) family problems (e.g., abuse, neglect, domestic violence); and (vi) housing instability. These psychosocial complexity factors were further investigated using associated sub-questions, including items querying the experiences of verbal, physical, and sexual abuse, as well as neglect; and experiences of criminal violence and family violence. ThYNC also included Section 2 (Items I, J, and K) of the Australian Treatment Outcome Profile (ATOP), a collection of questions designed to measure an individual’s level of psychological wellbeing, physical wellbeing, and quality of life on a standardized 10-point scale [32]. The ATOP shows strong validity and reliability in AOD samples [32].

### Procedure

The Youth Needs Census is a three-yearly activity in which all participating Victorian youth AOD sector workers complete a census survey on each client they are working with (most recently conducted in 2016 in Victoria). One survey was conducted on each young person enrolled in the youth AOD system on the census date. The ThYNC project received ethics approval from the Eastern Health Human Research Ethics Committee in 2013 (E28–1213) and 2016 (LR89/2016). All participants provided informed consent by reading a participant information and consent form before completing each survey online. Workers that did not provide informed consent were taken to an automatic thank you page and exited from the survey.

To complete ThYNC, all workers were informed of the upcoming census date and provided a link to an online survey offered using the Qualtrics software package (Qualtrics, Utah, USA). Managers were asked to monitor completion at their sites but records were not obtained on the number of surveys completed in each service to ensure the voluntary nature of the study for youth workers and services. The Youth Needs Census (2013 and 2016) was a 56-item clinician rated multiple choice audit tool [31]. ThYNC was completed by youth and health workers across Victorian Youth AOD services to report on every client in their caseload on a statewide census date. Services generated client lists on this nominated date (6 June in 2013 and 21 November in 2016) indicating a total eligible sample pool. A total of 36 services or service sites participated in 2013 and 28 services or service sites participated in 2016. Based on sample pool, 84% of eligible young people were reported on in 2013 and 96% in 2016. Upon completion, the Qualtrics database was exported to the Statistical Package for the Social Sciences (SPSS), Version 21. Cleaning and analysis of the data was then performed using SPSS.

### Data analysis strategy

Four variables were available as potential identifiers of problematic substance use: (i) daily or almost daily use; (ii) use within the past month; (iii) worker identification of young person as substance dependent; and (iv) worker identification of the substance of primary concern (i.e., treatment focus). Daily use, use in the past month, and dependence were coded yes or no, and were not mutually exclusive with other substances (e.g., an individual could be reported to use both cannabis and methamphetamine daily). Substance of primary concern was rated by substance, forming independent groups (e.g., cannabis, methamphetamines, or alcohol).

Daily or almost daily cannabis use was selected as the primary focus of hypothesis-testing, due to this variable most objectively capturing high frequency of use. Past month usage was judged to be less indicative of high frequency use, whilst dependence was not used as a measure as it was not determined using structured clinical tools and was not included in the 2013 ThYNC. The substance of primary concern variable was not included as it did not allow for acknowledgement of poly-substance use and was determined solely on clinician opinion. Past month usage, dependence, and substance of primary concern were however retained as informative in describing the sample.

Methamphetamines and alcohol were chosen for cross-substance comparison of psychosocial complexity factors. This was because cannabis, methamphetamines, and alcohol were the three most frequent substances of daily use in both the 2016 (54.2, 12.6, and 16.8% respectively) and 2013 samples (46.3, 10.4, and 17.8% respectively), excluding tobacco. Tobacco products were very commonly used (43.0% in 2016, 44.1% in 2013), but were not investigated in the present study as they were seldom identified as a primary concern (2.7% in 2016, 3.1% in 2013) in youth services clients. No other substance exceeded 5% of the sample in terms of daily use, dependence, or substance of primary concern.

The majority of ThYNC data was categorical in nature. For this reason, non-parametric testing was the most appropriate form of inferential statistics. Hypothesis-testing was conducted by generating contingency tables and deriving chi-square as the omnibus test, with responses of “other” or “unsure” excluded from analysis [33]. Cramer’s *V* was calculated to estimate effect size [33]. The significance threshold was set at *p* < .05. Bonferroni correction was not applied due to concerns that it would be overly conservative given the categorical nature of the data [34]. Initial descriptive statistics for the sample were run using daily use, past month use, dependence, and primary concern data as collected. Following this, in order to acknowledge poly-substance use and facilitate group comparison, the daily use variables for cannabis, methamphetamines, and alcohol were aggregated into a single variable containing five groups: (i) no daily substance use (*n* = 295); (ii) daily use of cannabis only (*n* = 309); (iii) daily use of cannabis as well as methamphetamines, alcohol, or all three substances (*n* = 137); (iv) daily use of methamphetamines only (*n* = 35); and (v) daily use of alcohol only (*n* = 41). Cases of daily use of both methamphetamines and alcohol (*n* = 6) were excluded as this substance combination was not a focus of the study and the group size was minuscule.

## Results

### Frequency of cannabis use

**Fig. 1:**
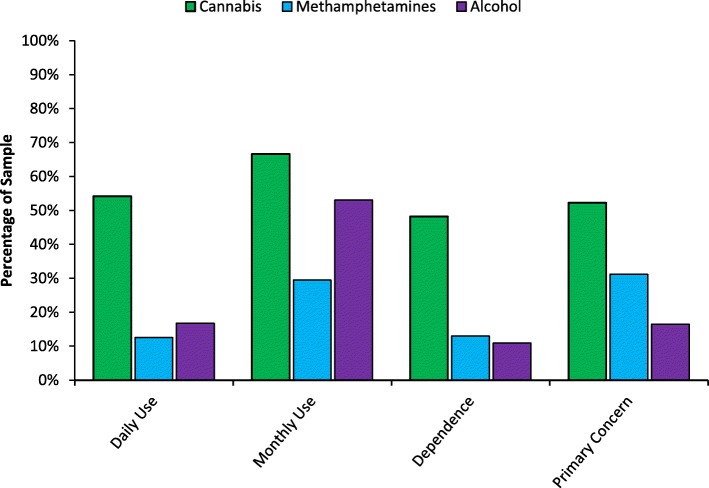
Case percentages for daily use, monthly use (past 4 weeks), dependence, and being the substance of primary concern across cannabis, methamphetamines, and alcohol

Figure 1 demonstrates that there were a greater proportion of daily cannabis use cases (54.2%) than daily methamphetamines (12.6%) or alcohol use cases (16.8%). Similarly, cases of past month cannabis use were more frequent (66.6%) than for methamphetamines (29.5%) or alcohol (53.1%). Youth workers indicated that 48.2% of the young people they were working with at the time of the census could be considered dependent on cannabis based on their clinical experience, and cannabis was the primary drug of concern in 52.3% of cases. This was a greater proportion of young clients than were dependent on methamphetamines or alcohol (13.0 and 10.9% respectively), or for whom methamphetamines or alcohol were the primary drug of concern (31.2 and 16.5% respectively). Daily cannabis use was not disproportionately represented by gender, Χ^2^(2) = 2.50, *p* = .287, Cramer’s *V* = .06, sexual orientation, Χ^2^(1) = 0.22, *p* = .639, Cramer’s *V* = .02, Aboriginal and Torres Strait Islander, Χ^2^(1) = 1.03, *p* = .310, Cramer’s *V* = .04, or asylum-seeker demographics, Χ^2^(1) = 0.48, *p* = .489, Cramer’s *V* = .02.

Overlap in daily or almost daily substance use was present, with 14.1% of young people engaging in daily cannabis also using methamphetamines daily, Spearman’s ρ(*n* = 823) = .05, *p* = .162, whilst 20.4% also used alcohol, Spearman’s ρ(*n* = 823) = .11, *p* = .002. In the past month, 33.0% of young people engaging in daily cannabis use had used methamphetamines, Spearman’s ρ(*n* = 823) = .08, *p* = .019, whilst 59.6% had also used alcohol, Spearman’s ρ(*n* = 823) = .14, *p* < .001. With regards to dependence, 84.8% of young people engaging in daily cannabis use were rated as dependent on cannabis, Spearman’s ρ(*n* = 823) = .80, *p* > .001, whilst 13.0% were dependent on methamphetamines, Spearman’s ρ(*n* = 823) < .01, *p* = .998, and 12.3% were dependent on alcohol, Spearman’s ρ(*n* = 823) = .05, *p* = .163. Cannabis was the primary substance of concern for 65.2% of young people engaging in daily cannabis use, with methamphetamines and alcohol the primary substance of concern for 19.1 and 7.2% respectively, Χ^2^(1) = 148.59, *p* > .001, Cramer’s *V* = .46.

### Comparison of substance use categories

**Fig. 2:**
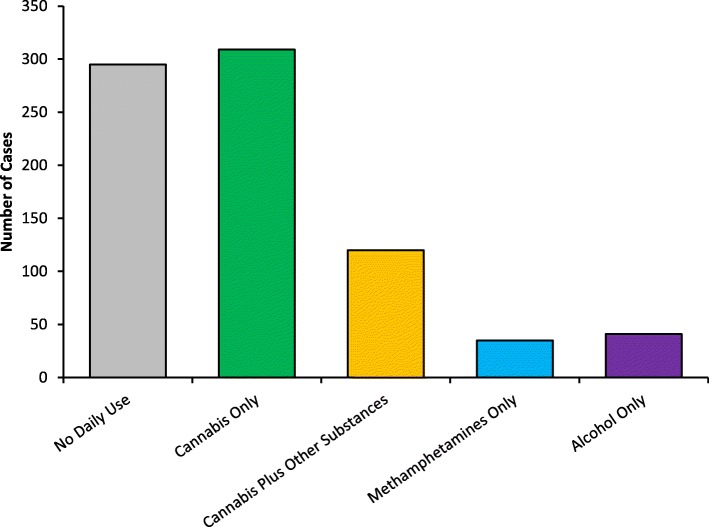
Separated case numbers for no daily substance use and daily use of cannabis, cannabis plus methamphetamines and/or alcohol, methamphetamines only, or alcohol only

**Fig. 3:**
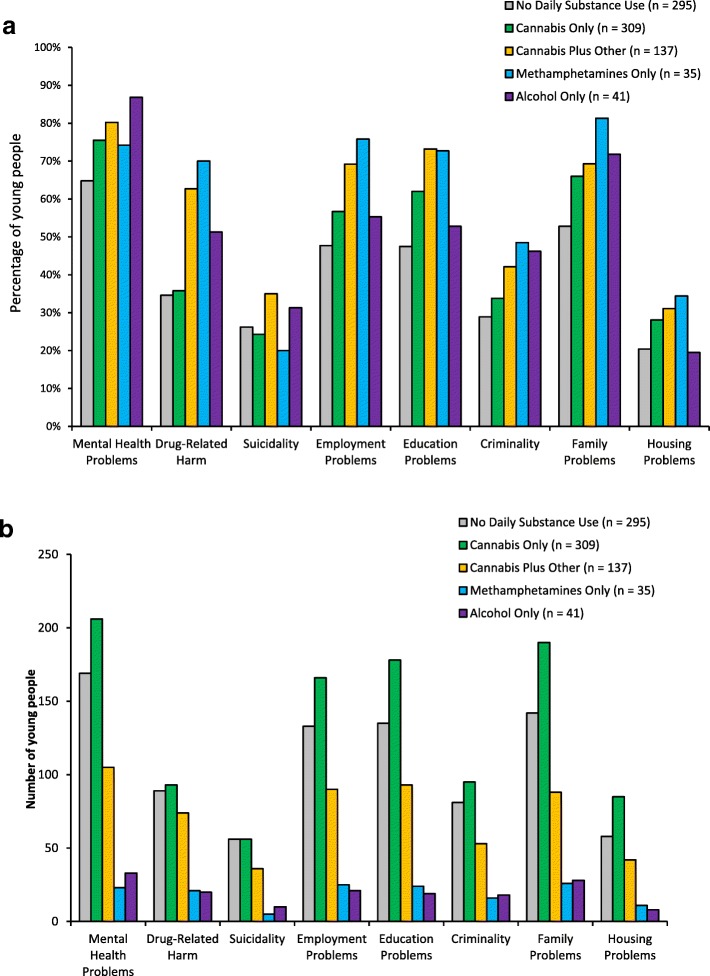
**a** Percentage of young people affected by psychosocial complexity factors across cases of no daily substance use and daily use of cannabis, cannabis plus methamphetamines and/or alcohol, methamphetamines only, or alcohol only. **b** Number of young people affected by psychosocial complexity factors across cases of no daily substance use and daily use of cannabis, cannabis plus methamphetamines and/or alcohol, methamphetamines only, or alcohol only

A visual comparison of group numbers is displayed in Fig. 2. There were significantly more cases of daily cannabis use than there were daily use cases of cannabis plus other substances, methamphetamines only, or alcohol only, Χ^2^(4) = 432.58, *p* > .001, supporting the first hypothesis. Figure 3a compares psychosocial complexity factors proportionately across the five substance use categories. Young people using cannabis daily were significantly more likely than young people not engaging in daily substance use to experience mental health problems, Χ^2^(1) = 7.32, *p* = .007, Cramer’s *V* = .12, employment problems, Χ^2^(1) = 4.63, *p* = .032, Cramer’s *V* = .09, education problems, Χ^2^(1) = 12.09, *p* = .001, Cramer’s *V* = .15, family problems, Χ^2^(1) = 10.04, *p* = .002, Cramer’s *V* = .13, and housing problems, Χ^2^(1) = 4.83, *p* = .028, Cramer’s *V* = .09, supporting the second hypothesis.

Notably, the rate of mental health problems in daily cannabis users included a 37.9% rate of formal diagnosis of a mental health condition. In terms of the past experiences of young people using cannabis daily, the data indicated that these young people had high lifetime histories of neglect (39.9%), emotional abuse (51.1%), physical abuse (39.5%), sexual abuse (15.5%) and being victims of violent crime (24.4%). In relation to family violence more specifically, young people using cannabis were reported to have witnessed family violence in 35.2% of cases, experienced as the victim of family violence in 34.3% and the instigator of family violence in 18.2% of cases. Daily cannabis use was weakly negatively associated with psychological wellbeing, Spearman’s ρ(*n* = 823) = −.17, *p* < .001, physical wellbeing, Spearman’s ρ(*n* = 823) = −.20, *p* < .001, and overall quality of life, Spearman’s ρ(*n* = 823) -.19, *p* < .001. Elaborating on the first hypothesis test, these data further indicate an association between cannabis use and poorer mental and physical health.

To test the third hypothesis, the proportion of each psychosocial complexity factor was compared across cases of daily cannabis use only, daily methamphetamine use only, and daily alcohol use only. No significant differences between the three substances were present for mental health problems, suicidality, employment problems, education problems, criminality, family problems, or housing problems, although young people engaging in daily use of cannabis only were significantly less likely to experience drug-related harm, Χ^2^(2) = 15.10, *p* = .001, Cramer’s *V* = .21. These results support the third hypothesis.

To acknowledge the occurrence of poly-substance use, where an individual may engage in the use of multiple substances on a daily basis, as well as the low number of young people using only methamphetamines or alcohol daily, cases of daily use of cannabis only were compared to cases of daily use of cannabis plus methamphetamines and/or alcohol in terms of psychosocial risk factors. Young people combining daily use of cannabis with daily use of methamphetamines and/or alcohol were significantly more likely to experience drug-related harm, Χ^2^(1) = 23.89, *p* < .001, Cramer’s *V* = .25, suicidality, Χ^2^(1) = 4.00, *p* < .045, Cramer’s *V* = .11, employment problems, Χ^2^(1) = 5.96, *p* < .015, Cramer’s *V* = .12, and education problems, Χ^2^(1) = 4.89, *p* < .027, Cramer’s *V* = .11. No significant differences were present for mental health problems, criminality, family problems, or housing problems.

The above hypothesis tests measured strength of association by analysing the proportion of psychosocial complexity factors relative to group size. This approach allowed inferences to be drawn regarding the likelihood of individual risk based on substance use category, but does not consider the greater prevalence of cannabis use. Figure 3b expresses the same data shown in Fig. 3a as number of participants, rather than as percentage of substance use group. Comparing the mean number of people impacted across the eight psychosocial complexity factors, the amount of young people using cannabis daily affected was 3.49:1 of those using methamphetamines daily, and 2.86:1 of those using alcohol daily, providing a metric of the broader social impact of cannabis use beyond individual risks.

### Comparison of 2016 and 2013 cohorts

The 2016 and 2013 cohorts did not significantly differ in age or gender. The proportion of young people using cannabis daily significantly increased to 54.2% in 2016 from 47.5% in 2013, Χ^2^(1) = 8.09, *p* = .004, Cramer’s *V* = .07. There were no significant changesin daily methamphetamine use or daily alcohol use between 2016 and 2013. Cannabis use in the past month remained stable between 2016 and 2013, however there were significantly less cases of use in the past month for both methampetamines, 29.5% versus 34.9% respectively, Χ^2^(1) = 5.95, *p* = .015, Cramer’s *V* = .06, and alcohol, 53.1% versus 63.1% respectively, Χ^2^(2) = 18.61, *p* < .001, Cramer’s *V* = .10. Dependence was not assessed in the 2013 sample. Cannabis was identified as the primary substance of concern in a greater proportion of cases in 2016, up to 52.3% in 2016 from 44.4% in 2013. Methamphetamines as primary concern increased only slightly between cohorts, 31.2% in 2016 versus 30.0% in 2013. The increase in young people for whom cannabis was a concern was a result of displacing individuals for whom alcohol was the primary concern, with alcohol as primary concern decreasing to 16.5% in 2016 from 25.6% in 2013. These frequency changes in primary substance of concern were significant, Χ^2^(2) = 19.56, *p* < .001, Cramer’s *V* = .11.

**Fig. 4:**
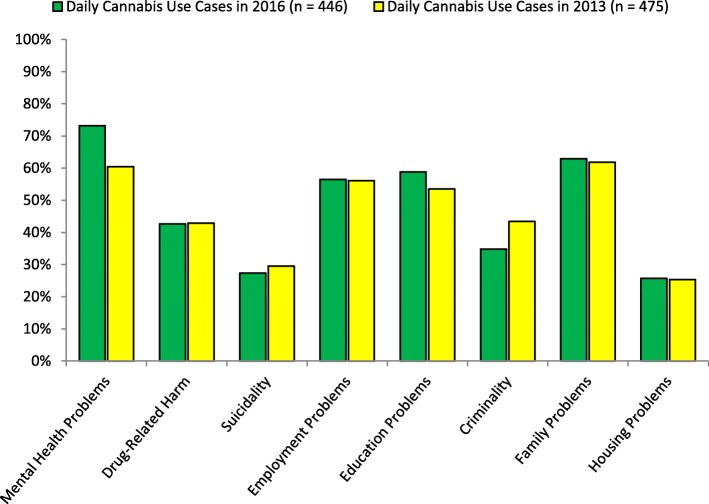
Comparison of cases of daily cannabis use experiencing psychosocial complexity factors across 2016 and 2013 cohorts

Investigating the fourth hypothesis, Fig. 4 compares young people using cannabis daily who were assessed in the 2016 ThYNC survey to those assessed in the 2013 ThYNC survey. Young people using cannabis daily in 2016 exhibited significantly higher rates of mental health problems, Χ^2^(1) = 29.44, *p* < .001, Cramer’s *V* = .13, and disengagement from education, Χ^2^(1) = 4.86, *p* = .028, Cramer’s *V* = .05, but a significantly lower rate of current criminal behaviour, Χ^2^(1) = 13.04, *p* < .001, Cramer’s *V* = .09. Rates of drug-related harm, suicide attempt presence, disengagement from employment, family problems, and housing problems remained stable between 2013 and 2016 in young people who were using cannabis daily.

## Discussion

This study examined cannabis use and psychosocial complexity factors in young people engaged with youth AOD services in 2016 and 2013. The results provide a picture of the impact that daily cannabis use has on young people accessing youth AOD services. As predicted in the first hypothesis, cannabis was found to be the most common substance of daily use, both on its own and in combination with other substances. Supporting the second hypothesis, cannabis use in young people was associated with elevated rates of mental health problems, disengagement from employment, disengagement from education, family problems, and housing problems. Mental health problems, suicidality, employment problems, education problems, criminality, family problems, and housing problems were present with daily cannabis use at a similar rate to their co-occurrence with daily methamphetamine and alcohol use, supporting the third hypothesis, although drug-related harm, suicidality, employment problems, and education problems were exacerbated when daily cannabis use was accompanied by daily use of another substance. In accord with the fourth hypothesis, the proportion of young people using cannabis daily had significantly increased between 2013 and 2016, although only mental health problems and disengagement from education worsened in frequency between 2013 and 2016.

The major finding of this study was that cannabis was the most frequent substance of daily use, monthly use, dependence, and primary concern in young people accessing youth AOD services. Rates of cannabis use were more than double the rates of methamphetamine and alcohol use in this sample, and this overwhelmingly higher prevalence of daily cannabis use was maintained even when daily use of cannabis and other substances was considered separately. These findings indicate that cannabis prevalence in individuals accessing youth AOD treatment has now overtaken that of alcohol, a legally accessible substance that was more commonly used than cannabis in the previous ThYNC census, and also show that despite widespread media coverage of methamphetamine problems [e.g., 35, 36], cannabis is a far more prevalent and problematic concern.

Daily cannabis use was associated with a range of psychosocial complexity factors, in a similar fashion that methamphetamines and alcohol were problematic for these young people. Only drug-related harm was proportionately more likely for young people using methamphetamines or alcohol daily as opposed to cannabis. Drug-related harm, suicidality, employment problems, and education problems were exacerbated when cannabis was used daily alongside other substances. Notably, these findings indicate that cannabis use in youths engaged with the youth AOD system in Victoria have significant negative personal, family and societal impact on young people. With half of youths engaged with youth AOD services using cannabis daily with the majority of these young people experiencing negative consequences, this highlights the complex social issue around effectively supporting young people with cannabis as the substance of concern. Indeed, when examined in terms of the number of people affected, individuals using cannabis daily constitute a significantly larger amount of young people experiencing psychosocial complexity factors than do those using alcohol or methamphetamines. Supporting young people presenting with daily cannabis use is therefore responsible for greater consumption of services.

Importantly, cannabis use is also an increasing concern, with daily use increasing by 6.7% between the 2013 and 2016 censuses, with the data suggesting that increasing cannabis use was displacing alcohol use in young people accessing youth services. The degree to which daily cannabis use was linked to the eight psychosocial risk factors examined by the present study largely remained stable since 2013. This means that for the past 3 years, most psychosocial concerns have not increased in prevalence in disadvantaged youth who use cannabis daily. However, both mental health problems and disengagement from education have worsened since 2013, with criminality lessening. It is timely when considering cannabis use and psychosocial complexity that ThYNC is an association study so there is no implication that increasing rates and level of cannabis use predict psychosocial complexity, nor that this complexity is predictive of cannabis use. Rather the study highlights to clinicians and researchers that there is often high rates of psychosocial complexity in young people engaged with AOD services who use cannabis on a daily basis [37]. This interdependency is expected when viewing substance use through the resilience [38] and developmental perspectives [39] adapted by youth AOD services where individual, family and systems concepts must be included into acre planning and treatment. .

The findings should be qualified in the context of limitations to the present research. Firstly, the categorical nature of the present data hindered parametric analysis. As a large survey that is sub-population-scale in scope, ThYNC questions are typically rated in dichotomous (yes-or-no) format, sometimes including an “other” or “unsure” option, with a focus on being as clearly defined and objective as possible for workers. This method of data collection meant that potentially useful quantitative metrics allowing for more rigorous statistical analysis (e.g., quantity of daily use, method of administration, etc.) were not available. Similarly, the large-scale nature of data collection for this study precluded the use of more involved standardised measures with established reliability and validity.

A second limitation was that the chi-square tests used to analyse the data identify an association, but do not assess cause. The eight psychosocial risk factors that were examined feasibly have a bidirectional relationship with substance use. Accordingly, these factors may have led to individuals seeking treatment from youth AOD services [40], or might have arisen from a common predisposing variable, such as a pre-existing predisposition to mental health problems [41]. In treating substance abuse, it is important to not just view these psychosocial complexity factors as probable outcomes, but to also acknowledge them as important precipitants that can be made a focus of treatment [15].

Thirdly, while not a limitation as such, it is important to qualify the scope of the present research. This study made use of two large-scale data sets recorded from youth AOD workers about the young people that they were working with. This means that the population being studied here are those accessing youth AOD services, with the large sample size and pattern of results indicating that cannabis use is an issue that should not be ignored in this population. However, these results may not generalise to the general population that is of interest when further quantifying the social impact of cannabis use. These data specifically relate to the issues surrounding youths who are presenting and engaging with youth AOD services, not a general measure of the impacts of cannabis youth in the general population. Whilst this limits generalizability to the wider youth community of Australia, this is deliberate focus. The results of both ThYNC surveys indicate that these young people are often disengaged with school and work, in unstable housing and experiencing other factors that make them less likely to be captured in large scale data collection efforts [e.g., 1, 2]. ThYNC acknowledges these young people are towards the edge of the typical pool of young people in terms of substance use and life circumstances (sometimes referred to as the “pointy end” of youth substance use) and that this survey methodology is deliberately developed to provide these young people with an advocacy and research voice. As the research only captured young people engaged with services, it is recognized that many more young people with similar levels of use do not engage with youth AOD services who do experience significant concerns. This highlights the need for services to be attuned and accessible to as many young people across the spectrum of substance use as possible.

Globally, mental, neurological and substance use disorders contribute to a significant proportion of disease burden [42]. However, data from the World Mental Health Surveys (WMHS) suggest that only a small minority (7.1%) of people with substance use disorders receive even minimally adequate treatment [43]. The present study has contributed to continuing debate about the importance of being aware of and complexity in managing cannabis use in young people. The findings underscore the need to remember that daily cannabis use is associated with a range of negative outcomes in these youths, and also that recovery from substance use disorders is highly possible made if people can access engaging and evidence-informed treatment services and social supports [44, 45]. Cannabis use is associated with detrimental psychosocial complexity factors to a similar extent as use of methamphetamines and alcohol, and the sheer prevalence of daily use makes cannabis a substance of concern from an epidemiological, social, personal and economic perspective.

## Conclusion

The association between regular cannabis use and psychosocial complexity has important clinical ramifications. For clients presenting with regular cannabis use, clinicians may benefit form exploring their experience of psychosocial complexity. Likewise, in people presenting with clear psychosocial complexity, screening for cannabis use may prove important and require consideration in the case formulation. Overall this research again highlights that systematic assessment of substance use in AOD help-seeking young people is important in any debate around cannabis, particularly those considering the issues from developmental and harm minimization perspectives.

## Data Availability

The datasets used and/or analysed during the current study are available from the corresponding author on reasonable request. Release of this dataset is currently restricted due to ongoing publications of materials from this larger study.

## Abbreviations

AOD: Alcohol and Other Drug
ThYNC: The Youth Needs Census
